# 3,4-Diamino­pyridinium 4-nitro­benzoate–4-nitro­benzoic acid (1/1)

**DOI:** 10.1107/S1600536809027354

**Published:** 2009-07-18

**Authors:** Hoong-Kun Fun, Kasthuri Balasubramani

**Affiliations:** a X-ray Crystallography Unit, School of Physics, Universiti Sains Malaysia, 11800 Universiti Sains Malaysia, Penang, Malaysia

## Abstract

In the title compound, C_5_H_8_N_3_^+^·C_7_H_4_NO_4_^−^·C_7_H_5_NO_4_, the non-H atoms of the 3,4-diamino­pyridinium cation are coplanar, with a maximum deviation of 0.022 (1) Å. The carboxyl­ate and nitro groups of the 4-nitro­benzoate anion are twisted out of the attached ring planes by dihedral angles of 15.89 (8) and 10.20 (8)°, respectively. In the 4-nitro­benzoic acid mol­ecule, the carboxyl and nitro groups form dihedral angles of 18.25 (8) and 6.55 (8)°, respectively, with the benzene ring. In the crystal, the constituent units form two-dimensional networks parallel to (001) by O—H⋯O, N—-H⋯O and C—H⋯O hydrogen bonds. Weak π–π inter­actions involving inversion-related 4-nitro­benzoic acid mol­ecules [centroid–centroid distance = 3.7325 (8) Å] and inversion-related 4-nitro­benzoate mol­ecules [centroid–centroid distance = 3.7124 (8) Å] are also observed.

## Related literature

For general background to substituted pyridines, see: Pozharski *et al.* (1997 ▶); Katritzky *et al.* (1996 ▶); For related structures, see: Opozda *et al.* (2006 ▶); Rubin-Preminger & Englert (2007 ▶); Koleva *et al.* (2007 ▶, 2008 ▶); Fun & Balasubramani (2009 ▶). For bond-length data, see: Allen *et al.* (1987 ▶). For hydrogen-bond motifs, see: Bernstein *et al.* (1995 ▶). For the stability of the temperature controller used in the data collection, see: Cosier & Glazer (1986 ▶).
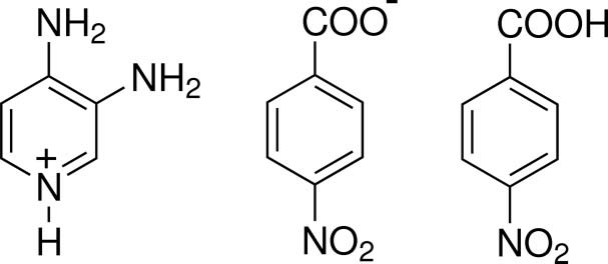


## Experimental

### 

#### Crystal data

C_5_H_8_N_3_^+^·C_7_H_4_NO_4_^−^·C_7_H_5_NO_4_
*M* *_r_* = 443.38
Triclinic, 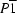

*a* = 6.8073 (2) Å
*b* = 6.8087 (2) Å
*c* = 21.0171 (5) Å
α = 80.859 (1)°
β = 83.253 (1)°
γ = 78.549 (1)°
*V* = 938.88 (5) Å^3^
*Z* = 2
Mo *K*α radiation
μ = 0.13 mm^−1^
*T* = 100 K
0.56 × 0.20 × 0.17 mm

#### Data collection

Bruker SMART APEXII CCD area-detector diffractometer
Absorption correction: multi-scan (*SADABS*; Bruker, 2005 ▶) *T*_min_ = 0.934, *T*_max_ = 0.979
27907 measured reflections
5435 independent reflections
4025 reflections with *I* > 2σ(*I*)
*R*_int_ = 0.041

#### Refinement

*R*[*F* ^2^ > 2σ(*F* ^2^)] = 0.059
*wR*(*F* ^2^) = 0.190
*S* = 1.05
5435 reflections
353 parameters
H atoms treated by a mixture of independent and constrained refinement
Δρ_max_ = 0.81 e Å^−3^
Δρ_min_ = −0.44 e Å^−3^

### 

Data collection: *APEX2* (Bruker, 2005 ▶); cell refinement: *SAINT* (Bruker, 2005 ▶); data reduction: *SAINT*; program(s) used to solve structure: *SHELXTL* (Sheldrick, 2008 ▶); program(s) used to refine structure: *SHELXTL*; molecular graphics: *SHELXTL* software used to prepare material for publication: *SHELXTL* and *PLATON* (Spek, 2009 ▶).

## Supplementary Material

Crystal structure: contains datablocks global, I. DOI: 10.1107/S1600536809027354/ci2849sup1.cif

Structure factors: contains datablocks I. DOI: 10.1107/S1600536809027354/ci2849Isup2.hkl

Additional supplementary materials:  crystallographic information; 3D view; checkCIF report

## Figures and Tables

**Table 1:** Hydrogen-bond geometry (Å, °)

*D*—H⋯*A*	*D*—H	H⋯*A*	*D*⋯*A*	*D*—H⋯*A*
O3*B*—H1*O*3⋯O3*A*^i^	0.82	1.65	2.463 (2)	173
N3—H1*N*3⋯O4*B*^ii^	1.05 (3)	2.08 (3)	3.008 (3)	146 (2)
N3—H2*N*3⋯O2*B*^iii^	0.92 (3)	2.39 (3)	3.129 (2)	138 (2)
N2—H1*N*2⋯O3*B*^iv^	1.00 (2)	2.00 (2)	2.929 (2)	154 (2)
N4—H1*N*4⋯O4*A*^i^	0.90 (2)	2.18 (2)	3.068 (2)	169 (2)
N4—H2*N*4⋯O3*A*^v^	0.89 (2)	2.35 (2)	3.152 (2)	150 (2)
C1*B*—H1*B*⋯O4*B*^ii^	0.94 (2)	2.52 (2)	3.231 (2)	133 (2)
C4*B*—H4*B*⋯O1*B*^vi^	0.97 (2)	2.54 (2)	3.250 (2)	130 (2)
C12—H12⋯O4*B*^vii^	0.89 (2)	2.50 (2)	3.376 (2)	165 (2)

Symmetry codes: (i) 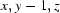; (ii) 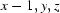; (iii) 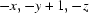; (iv) 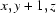; (v) 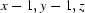; (vi) 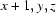; (vii) 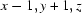.

**Fig. 1.**
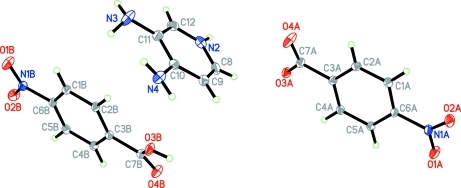
The asymmetric unit of the title compound, showing 50% probability displacement ellipsoids and the atom-numbering scheme.

**Fig. 2.**
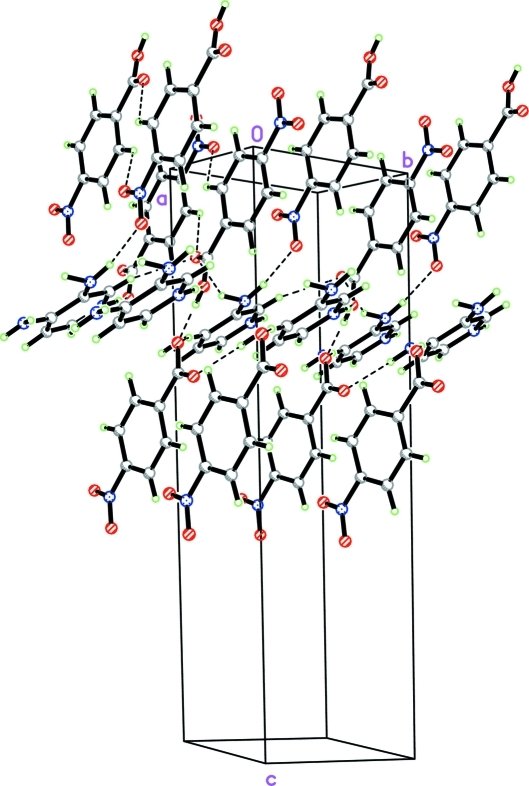
Part of the crystal packing in the title compound, showing a two-dimensional network parallel to the (001). Hydrogen bonds are shown as dashed lines.

## supplementary crystallographic information

### Comment

Pyridine and its derivatives play an important role in heterocyclic chemistry (Pozharski *et al.*, 1997; Katritzky *et al.*, 1996). 3,4-Diaminopyridine is used as a component in Schiff base reactions (Opozda *et al.*, 2006). The crystal structure of 3,4-diaminopyridine (Rubin-Preminger & Englert, 2007), 3,4-diaminopyridinium hydrogen squarate (Koleva *et al.*, 2007), 3,4-diaminopyridinium hydrogen tartarate (Koleva *et al.*, 2008) and 3,4-diaminopyridinium hydrogen succinate (Fun & Balasubramani, 2009) have been reported. Since our aim is to study some interesting hydrogen-bonding interactions, the synthesis and structure of the title compound, (I), is presented here.

The asymmetric unit of (I) contains a 3,4-diaminopyridinium cation, a 4-nitrobenzoate anion and a 4-nitrobenzoic acid molecule (Fig 1). The bond lengths (Allen *et al.*, 1987) and angles are normal.

In the 3,4-diaminopyridinium cation, the protonation of atom N2 has lead to a slight increase in C8—N2—C12 angle to 120.32 (15)° compared to 115.69 (19)° in 3,4-diaminopyridine (Rubin-Preminger & Englert, 2007). The non-H atoms of the 3,4-diaminopyridinium cation are coplanar, with a maximum deviation of 0.022 (1) Å for atom N4. The sum of bond angles associated with atoms N3 and N4 suggests that atom N3 is sp^3^ hybridized while atom N4 is sp^2^ hybridized.

In the 4-nitrobenzoate anion, the carboxylate group is twisted slightly from the attached ring; the dihedral angle between C1A-C6A and O3A/O4A/C3A/C7A planes is 15.89 (8)°. The nitro group is twisted away from the attached benzene ring by 10.20 (8)°. In the neutral 4-nitrobenzoic acid molecule, the carboxylic acid (O3B/O4b/C3B/C7B) and nitro (O1B/O2B/N1B/C6B) groups form dihedral angles of 18.25 (8)° and 6.55 (8)°, respectively, with the attached C1B-C6B benzene ring.

The dihedral angle between the benzene rings of 4-nitrobenzoate (C1A-C6A) anion and 4-nitrobenzoic acid (C1B-C6B) molecule is 6.16 (6)°. The pyridine ring (N2/C8-C12) forms dihedral angles of 71.75 (8)° and 65.83 (8)°, respectively, with the C1A-C6A and C1B-C6B rings.

In the crystal packing (Fig. 2), the two amino groups (N3 and N4) are involved in  N—H···O hydrogen bonding with two 4-nitrobenzoate O atoms (O3A and O4A), one 4-nitrobenzoic acid O atom (O4B) and with one nitro group O atom (O2B). The 4-nitrobenzoic acid hydrogen, H1O3, is hydrogen-bonded to the carboxylate oxygen atom of 4-nitrobenzoate through O—H···O bonds. The 4-nitrobenzoic acid carbon atoms (C1B & C4B) are involved in C—H···O hydrogen bonding with the carboxylic acid and nitro group O atoms O4B and O1B, to form an *R*_2_^2^(10) ring motif (Bernstein *et al.*, 1995). The O—H···O, N—H···O and C—H···O hydrogen bonds (Table 1) link all the constituent units to form a two-dimensional network parallel to the (001). The crystal structure is further stabilized by π-π interactions. The inversion related 4-nitrobenzoic acid molecules are stacked with a centroid-to-centroid distance of 3.7325 (8) Å. Similarly, the inversion related 4-nitrobenzoate molecules are stacked with a centroid-to-centroid distance of 3.7124 (8) Å.

### Experimental

Hot methanol solutions (20 ml) of 3,4-diaminopyridine (27 mg, Aldrich) and 4-nitrobenzoic acid (42 mg, Merck) were mixed and warmed over a heating magnetic stirrer for 5 minutes. The resulting solution was allowed to cool slowly at room temperature. Crystals of (I) appeared from the mother liquor after a few days.

### Refinement

All the H atoms (except carboxyl oxygen) were located from the difference Fourier map [N–H = 0.89 (2)–1.05 (3) Å, C–H = 0.89 (2)–1.00 (2) Å and allowed to refine freely. The oxygen H atom was positioned geometrically (O–H = 0.82 Å) and refined using a riding model *U*_iso_(H) = 1.5*U*_eq_(O).

### Crystal data

**Table:**

C_5_H_8_N_3_^+^·C_7_H_4_NO_4_^−^·C_7_H_5_NO_4_	*Z* = 2
*M**_r_* = 443.38	*F*(000) = 460
Triclinic, *P*1	*D*_x_ = 1.568 Mg m^−^^3^
Hall symbol: -P 1	Mo *K*α radiation, λ = 0.71073 Å
*a* = 6.8073 (2) Å	Cell parameters from 7405 reflections
*b* = 6.8087 (2) Å	θ = 3.1–33.7°
*c* = 21.0171 (5) Å	µ = 0.13 mm^−^^1^
α = 80.859 (1)°	*T* = 100 K
β = 83.253 (1)°	Block, yellow
γ = 78.549 (1)°	0.56 × 0.20 × 0.17 mm
*V* = 938.88 (5) Å^3^	

### Data collection

**Table:**

Bruker SMART APEXII CCD area-detector diffractometer	5435 independent reflections
Radiation source: fine-focus sealed tube	4025 reflections with *I* > 2σ(*I*)
graphite	*R*_int_ = 0.041
φ and ω scans	θ_max_ = 30.0°, θ_min_ = 1.0°
Absorption correction: multi-scan (*SADABS*; Bruker, 2005)	*h* = −9→9
*T*_min_ = 0.934, *T*_max_ = 0.979	*k* = −9→9
27907 measured reflections	*l* = −29→28

### Refinement

**Table:**

Refinement on *F*^2^	Primary atom site location: structure-invariant direct methods
Least-squares matrix: full	Secondary atom site location: difference Fourier map
*R*[*F*^2^ > 2σ(*F*^2^)] = 0.059	Hydrogen site location: inferred from neighbouring sites
*wR*(*F*^2^) = 0.190	H atoms treated by a mixture of independent and constrained refinement
*S* = 1.05	*w* = 1/[σ^2^(*F*_o_^2^) + (0.1091*P*)^2^ + 0.3802*P*] where *P* = (*F*_o_^2^ + 2*F*_c_^2^)/3
5435 reflections	(Δ/σ)_max_ = 0.001
353 parameters	Δρ_max_ = 0.81 e Å^−^^3^
0 restraints	Δρ_min_ = −0.44 e Å^−^^3^

### Special details

**Table:**

Experimental. The crystal was placed in the cold stream of an Oxford Cyrosystems Cobra open-flow nitrogen cryostat (Cosier & Glazer, 1986) operating at 100.0 (1) K.
Geometry. All s.u.'s (except the s.u. in the dihedral angle between two l.s. planes) are estimated using the full covariance matrix. The cell s.u.'s are taken into account individually in the estimation of s.u.'s in distances, angles and torsion angles; correlations between s.u.'s in cell parameters are only used when they are defined by crystal symmetry. An approximate (isotropic) treatment of cell s.u.'s is used for estimating s.u.'s involving l.s. planes.
Refinement. Refinement of F^2^ against ALL reflections. The weighted R-factor wR and goodness of fit S are based on F^2^, conventional R-factors R are based on F, with F set to zero for negative F^2^. The threshold expression of F^2^ > σ(F^2^) is used only for calculating R-factors(gt) etc. and is not relevant to the choice of reflections for refinement. R-factors based on F^2^ are statistically about twice as large as those based on F, and R- factors based on ALL data will be even larger.

### Fractional atomic coordinates and isotropic or equivalent isotropic displacement parameters (Å^2^)

**Table:**

	*x*	*y*	*z*	*U*_iso_*/*U*_eq_	
O1A	1.39968 (18)	0.6458 (2)	0.56237 (6)	0.0292 (3)	
O2A	1.1299 (2)	0.6247 (2)	0.62692 (6)	0.0282 (3)	
O3A	0.84687 (17)	0.9461 (2)	0.30121 (6)	0.0235 (3)	
O4A	0.56283 (19)	0.8966 (2)	0.36256 (7)	0.0342 (4)	
C7A	0.7456 (2)	0.8934 (2)	0.35545 (8)	0.0193 (3)	
C1A	0.8875 (2)	0.7614 (2)	0.52810 (8)	0.0175 (3)	
C2A	0.7744 (2)	0.8188 (2)	0.47485 (8)	0.0178 (3)	
C3A	0.8705 (2)	0.8287 (2)	0.41221 (7)	0.0160 (3)	
C4A	1.0802 (2)	0.7779 (2)	0.40257 (8)	0.0173 (3)	
C5A	1.1946 (2)	0.7216 (2)	0.45525 (7)	0.0166 (3)	
C6A	1.0950 (2)	0.7158 (2)	0.51679 (7)	0.0155 (3)	
N1A	1.2168 (2)	0.6579 (2)	0.57258 (7)	0.0186 (3)	
O1B	0.08979 (19)	0.3360 (2)	−0.05503 (7)	0.0309 (3)	
O2B	0.3609 (2)	0.3677 (2)	−0.11725 (6)	0.0285 (3)	
O3B	0.64656 (17)	0.0180 (2)	0.20718 (6)	0.0239 (3)	
H1O3	0.7184	0.0014	0.2370	0.036*	
O4B	0.91654 (18)	0.1156 (2)	0.14870 (6)	0.0276 (3)	
C7B	0.7393 (2)	0.0931 (2)	0.15458 (8)	0.0185 (3)	
C1B	0.2910 (2)	0.2478 (2)	0.05366 (8)	0.0177 (3)	
C2B	0.4051 (2)	0.1875 (2)	0.10658 (7)	0.0170 (3)	
C3B	0.6149 (2)	0.1559 (2)	0.09760 (8)	0.0164 (3)	
C4B	0.7116 (2)	0.1852 (2)	0.03518 (8)	0.0180 (3)	
C5B	0.5997 (2)	0.2440 (2)	−0.01814 (8)	0.0177 (3)	
C6B	0.3922 (2)	0.2731 (2)	−0.00739 (7)	0.0163 (3)	
N1B	0.2727 (2)	0.3303 (2)	−0.06376 (7)	0.0188 (3)	
N2	0.3773 (2)	0.7229 (2)	0.23610 (7)	0.0261 (3)	
N3	−0.0247 (2)	0.4644 (3)	0.21167 (9)	0.0355 (4)	
N4	0.2251 (2)	0.1578 (2)	0.28541 (7)	0.0254 (3)	
C8	0.4928 (3)	0.5748 (3)	0.27289 (9)	0.0243 (4)	
C9	0.4450 (3)	0.3893 (3)	0.28994 (9)	0.0242 (4)	
C10	0.2719 (2)	0.3463 (3)	0.27034 (8)	0.0204 (3)	
C11	0.1493 (2)	0.5011 (3)	0.23126 (8)	0.0229 (3)	
C12	0.2073 (3)	0.6893 (3)	0.21562 (9)	0.0241 (4)	
H1N3	−0.023 (4)	0.312 (4)	0.2071 (13)	0.048 (7)*	
H2N3	−0.080 (4)	0.564 (4)	0.1805 (15)	0.060 (8)*	
H1N2	0.431 (3)	0.851 (4)	0.2220 (12)	0.036 (6)*	
H1N4	0.312 (3)	0.070 (3)	0.3102 (11)	0.028 (5)*	
H2N4	0.098 (3)	0.146 (3)	0.2834 (11)	0.028 (6)*	
H1A	0.820 (3)	0.765 (3)	0.5715 (11)	0.027 (5)*	
H2A	0.630 (4)	0.852 (3)	0.4818 (11)	0.034 (6)*	
H4A	1.145 (3)	0.772 (3)	0.3597 (11)	0.023 (5)*	
H5A	1.333 (3)	0.687 (3)	0.4501 (11)	0.027 (5)*	
H8A	0.599 (4)	0.613 (4)	0.2850 (14)	0.052 (8)*	
H1B	0.150 (4)	0.265 (3)	0.0608 (11)	0.033 (6)*	
H2B	0.340 (3)	0.173 (3)	0.1505 (11)	0.029 (6)*	
H4B	0.857 (4)	0.163 (4)	0.0290 (12)	0.036 (6)*	
H5B	0.668 (4)	0.250 (4)	−0.0633 (12)	0.036 (6)*	
H9	0.524 (4)	0.285 (4)	0.3184 (12)	0.041 (7)*	
H12	0.147 (3)	0.800 (4)	0.1914 (12)	0.034 (6)*	

### Atomic displacement parameters (Å^2^)

**Table:**

	*U*^11^	*U*^22^	*U*^33^	*U*^12^	*U*^13^	*U*^23^
O1A	0.0199 (6)	0.0440 (8)	0.0234 (7)	−0.0055 (5)	−0.0076 (5)	−0.0003 (6)
O2A	0.0303 (7)	0.0390 (7)	0.0133 (6)	−0.0033 (5)	−0.0016 (5)	−0.0015 (5)
O3A	0.0195 (6)	0.0354 (7)	0.0147 (6)	−0.0023 (5)	−0.0024 (4)	−0.0030 (5)
O4A	0.0216 (6)	0.0494 (8)	0.0304 (7)	−0.0152 (6)	−0.0123 (5)	0.0159 (6)
C7A	0.0211 (7)	0.0166 (7)	0.0216 (8)	−0.0049 (6)	−0.0082 (6)	−0.0007 (6)
C1A	0.0199 (7)	0.0178 (7)	0.0150 (7)	−0.0043 (5)	−0.0003 (6)	−0.0026 (5)
C2A	0.0156 (7)	0.0178 (7)	0.0201 (8)	−0.0033 (5)	−0.0021 (6)	−0.0022 (6)
C3A	0.0173 (7)	0.0151 (6)	0.0158 (7)	−0.0034 (5)	−0.0034 (5)	−0.0010 (5)
C4A	0.0194 (7)	0.0183 (7)	0.0140 (7)	−0.0032 (5)	−0.0020 (5)	−0.0018 (5)
C5A	0.0156 (7)	0.0186 (7)	0.0158 (7)	−0.0029 (5)	−0.0015 (5)	−0.0029 (5)
C6A	0.0178 (7)	0.0154 (6)	0.0138 (7)	−0.0029 (5)	−0.0043 (5)	−0.0017 (5)
N1A	0.0223 (7)	0.0190 (6)	0.0151 (6)	−0.0033 (5)	−0.0047 (5)	−0.0026 (5)
O1B	0.0201 (6)	0.0475 (8)	0.0244 (7)	−0.0067 (5)	−0.0078 (5)	0.0025 (6)
O2B	0.0286 (6)	0.0405 (7)	0.0124 (6)	0.0004 (5)	−0.0014 (5)	−0.0008 (5)
O3B	0.0206 (6)	0.0375 (7)	0.0136 (6)	−0.0041 (5)	−0.0057 (4)	−0.0020 (5)
O4B	0.0197 (6)	0.0396 (7)	0.0237 (6)	−0.0069 (5)	−0.0077 (5)	0.0014 (5)
C7B	0.0193 (7)	0.0195 (7)	0.0172 (7)	−0.0010 (6)	−0.0052 (6)	−0.0042 (6)
C1B	0.0146 (7)	0.0212 (7)	0.0179 (7)	−0.0024 (5)	−0.0019 (5)	−0.0050 (6)
C2B	0.0182 (7)	0.0206 (7)	0.0125 (7)	−0.0033 (5)	−0.0023 (5)	−0.0034 (5)
C3B	0.0173 (7)	0.0165 (7)	0.0158 (7)	−0.0025 (5)	−0.0038 (5)	−0.0028 (5)
C4B	0.0164 (7)	0.0206 (7)	0.0174 (7)	−0.0028 (5)	−0.0022 (5)	−0.0040 (6)
C5B	0.0199 (7)	0.0189 (7)	0.0143 (7)	−0.0040 (5)	−0.0011 (5)	−0.0025 (5)
C6B	0.0185 (7)	0.0170 (7)	0.0137 (7)	−0.0025 (5)	−0.0047 (5)	−0.0019 (5)
N1B	0.0214 (6)	0.0186 (6)	0.0161 (6)	−0.0015 (5)	−0.0054 (5)	−0.0021 (5)
N2	0.0330 (8)	0.0230 (7)	0.0230 (8)	−0.0063 (6)	0.0024 (6)	−0.0071 (6)
N3	0.0229 (8)	0.0494 (11)	0.0333 (9)	−0.0102 (7)	−0.0129 (7)	0.0087 (8)
N4	0.0211 (7)	0.0290 (8)	0.0251 (8)	−0.0062 (6)	−0.0041 (6)	0.0025 (6)
C8	0.0315 (9)	0.0230 (8)	0.0215 (8)	−0.0065 (7)	−0.0059 (7)	−0.0080 (6)
C9	0.0279 (8)	0.0240 (8)	0.0204 (8)	−0.0003 (7)	−0.0063 (6)	−0.0049 (6)
C10	0.0203 (7)	0.0244 (8)	0.0156 (7)	−0.0019 (6)	0.0019 (6)	−0.0057 (6)
C11	0.0168 (7)	0.0332 (9)	0.0177 (8)	−0.0018 (6)	0.0002 (6)	−0.0048 (6)
C12	0.0248 (8)	0.0243 (8)	0.0202 (8)	0.0018 (6)	0.0007 (6)	−0.0036 (6)

### Geometric parameters (Å, °)

**Table:**

O1A—N1A	1.2269 (18)	C2B—C3B	1.396 (2)
O2A—N1A	1.2317 (18)	C2B—H2B	0.98 (2)
O3A—C7A	1.299 (2)	C3B—C4B	1.400 (2)
O4A—C7A	1.232 (2)	C4B—C5B	1.390 (2)
C7A—C3A	1.504 (2)	C4B—H4B	0.97 (2)
C1A—C6A	1.386 (2)	C5B—C6B	1.384 (2)
C1A—C2A	1.393 (2)	C5B—H5B	1.00 (2)
C1A—H1A	0.97 (2)	C6B—N1B	1.473 (2)
C2A—C3A	1.397 (2)	N2—C8	1.347 (2)
C2A—H2A	0.96 (2)	N2—C12	1.353 (2)
C3A—C4A	1.399 (2)	N2—H1N2	1.00 (2)
C4A—C5A	1.389 (2)	N3—C11	1.379 (2)
C4A—H4A	0.96 (2)	N3—H1N3	1.05 (3)
C5A—C6A	1.386 (2)	N3—H2N3	0.92 (3)
C5A—H5A	0.92 (2)	N4—C10	1.364 (2)
C6A—N1A	1.4727 (19)	N4—H1N4	0.90 (2)
O1B—N1B	1.2307 (18)	N4—H2N4	0.89 (2)
O2B—N1B	1.2247 (18)	C8—C9	1.349 (2)
O3B—C7B	1.2913 (19)	C8—H8A	0.89 (3)
O3B—H1O3	0.82	C9—C10	1.390 (2)
O4B—C7B	1.2355 (19)	C9—H9	0.97 (2)
C7B—C3B	1.505 (2)	C10—C11	1.421 (2)
C1B—C6B	1.386 (2)	C11—C12	1.394 (3)
C1B—C2B	1.393 (2)	C12—H12	0.89 (2)
C1B—H1B	0.94 (2)		
			
O4A—C7A—O3A	125.20 (15)	C5B—C4B—C3B	120.32 (14)
O4A—C7A—C3A	120.56 (15)	C5B—C4B—H4B	119.7 (14)
O3A—C7A—C3A	114.22 (13)	C3B—C4B—H4B	120.0 (14)
C6A—C1A—C2A	118.09 (14)	C6B—C5B—C4B	118.05 (14)
C6A—C1A—H1A	122.4 (13)	C6B—C5B—H5B	121.0 (14)
C2A—C1A—H1A	119.3 (13)	C4B—C5B—H5B	120.6 (14)
C1A—C2A—C3A	120.10 (14)	C5B—C6B—C1B	123.32 (14)
C1A—C2A—H2A	119.2 (14)	C5B—C6B—N1B	118.39 (14)
C3A—C2A—H2A	120.7 (14)	C1B—C6B—N1B	118.28 (13)
C2A—C3A—C4A	120.26 (14)	O2B—N1B—O1B	123.15 (14)
C2A—C3A—C7A	119.19 (14)	O2B—N1B—C6B	118.26 (13)
C4A—C3A—C7A	120.55 (14)	O1B—N1B—C6B	118.58 (13)
C5A—C4A—C3A	120.19 (14)	C8—N2—C12	120.32 (15)
C5A—C4A—H4A	119.4 (12)	C8—N2—H1N2	116.5 (14)
C3A—C4A—H4A	120.3 (12)	C12—N2—H1N2	122.9 (14)
C6A—C5A—C4A	118.16 (14)	C11—N3—H1N3	114.8 (14)
C6A—C5A—H5A	120.1 (14)	C11—N3—H2N3	112.8 (18)
C4A—C5A—H5A	121.7 (14)	H1N3—N3—H2N3	119 (2)
C1A—C6A—C5A	123.18 (14)	C10—N4—H1N4	113.8 (14)
C1A—C6A—N1A	118.74 (13)	C10—N4—H2N4	117.7 (14)
C5A—C6A—N1A	118.08 (13)	H1N4—N4—H2N4	124 (2)
O1A—N1A—O2A	123.50 (14)	N2—C8—C9	121.67 (17)
O1A—N1A—C6A	118.07 (13)	N2—C8—H8A	113.6 (18)
O2A—N1A—C6A	118.43 (13)	C9—C8—H8A	124.7 (18)
C7B—O3B—H1O3	109.5	C8—C9—C10	120.49 (16)
O4B—C7B—O3B	125.03 (14)	C8—C9—H9	121.6 (14)
O4B—C7B—C3B	119.89 (14)	C10—C9—H9	117.7 (14)
O3B—C7B—C3B	115.08 (13)	N4—C10—C9	121.17 (15)
C6B—C1B—C2B	117.96 (14)	N4—C10—C11	120.42 (16)
C6B—C1B—H1B	123.3 (15)	C9—C10—C11	118.34 (16)
C2B—C1B—H1B	118.7 (15)	N3—C11—C12	121.98 (16)
C1B—C2B—C3B	120.33 (14)	N3—C11—C10	119.85 (16)
C1B—C2B—H2B	120.5 (13)	C12—C11—C10	118.13 (16)
C3B—C2B—H2B	119.1 (13)	N2—C12—C11	121.03 (16)
C2B—C3B—C4B	120.01 (14)	N2—C12—H12	110.3 (14)
C2B—C3B—C7B	120.67 (14)	C11—C12—H12	128.6 (15)
C4B—C3B—C7B	119.31 (13)		
			
C6A—C1A—C2A—C3A	0.1 (2)	O3B—C7B—C3B—C4B	−162.32 (14)
C1A—C2A—C3A—C4A	1.0 (2)	C2B—C3B—C4B—C5B	−0.6 (2)
C1A—C2A—C3A—C7A	−179.27 (14)	C7B—C3B—C4B—C5B	−179.72 (14)
O4A—C7A—C3A—C2A	−15.2 (2)	C3B—C4B—C5B—C6B	0.3 (2)
O3A—C7A—C3A—C2A	163.72 (14)	C4B—C5B—C6B—C1B	0.4 (2)
O4A—C7A—C3A—C4A	164.48 (16)	C4B—C5B—C6B—N1B	−178.44 (13)
O3A—C7A—C3A—C4A	−16.6 (2)	C2B—C1B—C6B—C5B	−0.8 (2)
C2A—C3A—C4A—C5A	−1.4 (2)	C2B—C1B—C6B—N1B	178.02 (13)
C7A—C3A—C4A—C5A	178.91 (14)	C5B—C6B—N1B—O2B	−6.6 (2)
C3A—C4A—C5A—C6A	0.6 (2)	C1B—C6B—N1B—O2B	174.52 (14)
C2A—C1A—C6A—C5A	−1.0 (2)	C5B—C6B—N1B—O1B	172.62 (14)
C2A—C1A—C6A—N1A	179.05 (13)	C1B—C6B—N1B—O1B	−6.3 (2)
C4A—C5A—C6A—C1A	0.7 (2)	C12—N2—C8—C9	0.6 (3)
C4A—C5A—C6A—N1A	−179.40 (13)	N2—C8—C9—C10	−0.7 (3)
C1A—C6A—N1A—O1A	−169.88 (14)	C8—C9—C10—N4	177.85 (16)
C5A—C6A—N1A—O1A	10.2 (2)	C8—C9—C10—C11	1.0 (3)
C1A—C6A—N1A—O2A	10.0 (2)	N4—C10—C11—N3	4.0 (3)
C5A—C6A—N1A—O2A	−169.97 (14)	C9—C10—C11—N3	−179.15 (16)
C6B—C1B—C2B—C3B	0.5 (2)	N4—C10—C11—C12	−178.05 (16)
C1B—C2B—C3B—C4B	0.2 (2)	C9—C10—C11—C12	−1.2 (2)
C1B—C2B—C3B—C7B	179.27 (14)	C8—N2—C12—C11	−0.8 (3)
O4B—C7B—C3B—C2B	−161.34 (15)	N3—C11—C12—N2	179.05 (17)
O3B—C7B—C3B—C2B	18.6 (2)	C10—C11—C12—N2	1.1 (2)
O4B—C7B—C3B—C4B	17.8 (2)		

### Hydrogen-bond geometry (Å, °)

**Table:**

*D*—H···*A*	*D*—H	H···*A*	*D*···*A*	*D*—H···*A*
O3B—H1O3···O3A^i^	0.82	1.65	2.463 (2)	173
N3—H1N3···O4B^ii^	1.05 (3)	2.08 (3)	3.008 (3)	146 (2)
N3—H2N3···O2B^iii^	0.92 (3)	2.39 (3)	3.129 (2)	138 (2)
N2—H1N2···O3B^iv^	1.00 (2)	2.00 (2)	2.929 (2)	154 (2)
N4—H1N4···O4A^i^	0.90 (2)	2.18 (2)	3.068 (2)	169 (2)
N4—H2N4···O3A^v^	0.89 (2)	2.35 (2)	3.152 (2)	150 (2)
C1B—H1B···O4B^ii^	0.94 (2)	2.52 (2)	3.231 (2)	133 (2)
C4B—H4B···O1B^vi^	0.97 (2)	2.54 (2)	3.250 (2)	130 (2)
C12—H12···O4B^vii^	0.89 (2)	2.50 (2)	3.376 (2)	165 (2)

Symmetry codes: (i) *x*, *y*−1, *z*; (ii) *x*−1, *y*, *z*; (iii) −*x*, −*y*+1, −*z*; (iv) *x*, *y*+1, *z*; (v) *x*−1, *y*−1, *z*; (vi) *x*+1, *y*, *z*; (vii) *x*−1, *y*+1, *z*.
